# Overcoming cold tumors: a combination strategy of immune checkpoint inhibitors

**DOI:** 10.3389/fimmu.2024.1344272

**Published:** 2024-03-13

**Authors:** Peng Ouyang, Lijuan Wang, Jianlong Wu, Yao Tian, Caiyun Chen, Dengsheng Li, Zengxi Yao, Ruichang Chen, Guoan Xiang, Jin Gong, Zhen Bao

**Affiliations:** ^1^ Department of General Surgery, The First Affiliated Hospital of Jinan University, Guangzhou, Guangdong, China; ^2^ Department of Pathophysiology, School of Medicine, Jinan University, Guangzhou, Guangdong, China; ^3^ Department of General Surgery, Guangdong Second Provincial General Hospital, Guangzhou, Guangdong, China

**Keywords:** cold tumors, immune checkpoint inhibitors, tumor-infiltrating T lymphocytes, tumor microenvironment, immunotherapy

## Abstract

Immune Checkpoint Inhibitors (ICIs) therapy has advanced significantly in treating malignant tumors, though most ‘cold’ tumors show no response. This resistance mainly arises from the varied immune evasion mechanisms. Hence, understanding the transformation from ‘cold’ to ‘hot’ tumors is essential in developing effective cancer treatments. Furthermore, tumor immune profiling is critical, requiring a range of diagnostic techniques and biomarkers for evaluation. The success of immunotherapy relies on T cells’ ability to recognize and eliminate tumor cells. In ‘cold’ tumors, the absence of T cell infiltration leads to the ineffectiveness of ICI therapy. Addressing these challenges, especially the impairment in T cell activation and homing, is crucial to enhance ICI therapy’s efficacy. Concurrently, strategies to convert ‘cold’ tumors into ‘hot’ ones, including boosting T cell infiltration and adoptive therapies such as T cell-recruiting bispecific antibodies and Chimeric Antigen Receptor (CAR) T cells, are under extensive exploration. Thus, identifying key factors that impact tumor T cell infiltration is vital for creating effective treatments targeting ‘cold’ tumors.

## 1 Introduction

In recent years, Immune Checkpoint Inhibitors (ICIs) have increasingly been incorporated into the treatment of various cancers, becoming a standard part of oncological treatment guidelines. However, a significant proportion of cancer patients still exhibit poor responses to ICI therapy. This trend highlights a need for further research and development in personalized cancer treatment strategies to improve outcomes for this patient subset (1, 2). In patients with solid tumors, ‘hot’ tumors (‘immune- inflamed’) often show a favorable response to ICIs, characterized by extensive lymphocyte infiltration in the tumor parenchyma. In contrast, ‘cold’ tumors exhibit a poorer response to ICIs. These tumors are marked by an inability of T cells to penetrate the tumor parenchyma, remaining instead in the tumor stroma (‘immune-excluded’) or by a lack of T cell infiltration in both the tumor parenchyma and stroma (‘immune-desert’) (3). This distinction underscores the importance of understanding tumor immunology to optimize ICIs therapy efficacy. However, increasing evidence suggests that not all tumors with high T cell infiltration exhibit favorable responses to ICIs. Conversely, some tumors with low T cell infiltration may also demonstrate good responsiveness to ICIs. This observation indicates a more complex relationship between T cell infiltration levels and ICI response, underscoring the need for a deeper understanding of tumor immunobiology to effectively predict and enhance ICIs therapy outcomes (4–6). These findings indicate that T cell infiltration might be necessary, but additional factors may be required for precisely identifying the responsiveness to ICIs. Currently, the treatment of ‘cold’ tumors remains a significant challenge. In this review, we discuss the definitions of ‘cold’ and ‘hot’ tumors, as well as the challenges the immune system may encounter at different stages of the cancer immunity cycle. We also describe therapeutic approaches combining ICIs with other strategies to overcome ‘cold’ tumors. This integrative approach aims to enhance the understanding and treatment efficacy of tumors with varying immune characteristics.

## 2 Definition of “cold” and “hot” tumors

The concept of ‘cold’ and ‘hot’ tumors is not new in the field of oncology. It was first described in 2006 by Galon et al. in their publication on the relationship between immune cell types, density, and distribution with the prognosis of colorectal cancer. This seminal work introduced the idea of classifying tumors as ‘hot’ or ‘cold’ based on the type, density, and distribution of immune cells within the tumor microenvironment. They posited that this immune-based classification in colorectal cancer could provide a more accurate prognosis assessment than the traditional TNM staging system. This approach underlines the significant role of the immune landscape in understanding and predicting cancer progression (7). In 2007, Galon and colleagues proposed the concept of “immune contexture” based on immunoscore (8). Following this, in 2009, Camus et al. first described three immune coordination profiles (hot, altered, and cold) in primary colorectal cancer (CRC), balancing tumor escape and immune coordination (9). Building on these works, researchers introduced the immunoscore, which assesses the infiltration of lymphocyte populations (CD3 and CD8) in the tumor core and at its margin. The score ranges from immunoscore 0 (I0, low-density CD3 and CD8 stained cells in the tumor center and periphery) to immunoscore 4 (I4, high-density CD3 and CD8 stained cells in these regions) (10, 11). This scoring system classifies cancer based on immune infiltration and introduces the concepts of ‘hot’ tumors (I4) and ‘cold’ tumors (I0-I3). As research progressed, the characteristics of hot tumors were expanded to include the presence of tumor-infiltrating lymphocytes (TILs), expression of programmed death-ligand 1 (PD-L1) on tumor-associated immune cells, and a high tumor mutational burden. Conversely, cold tumors, characterized by poor infiltration, also feature low or negligible PD-L1 expression, high proliferation rates, and a low tumor mutational burden (12).

## 3 Mechanisms of immune escape in “cold” tumors

**Figure 1:**
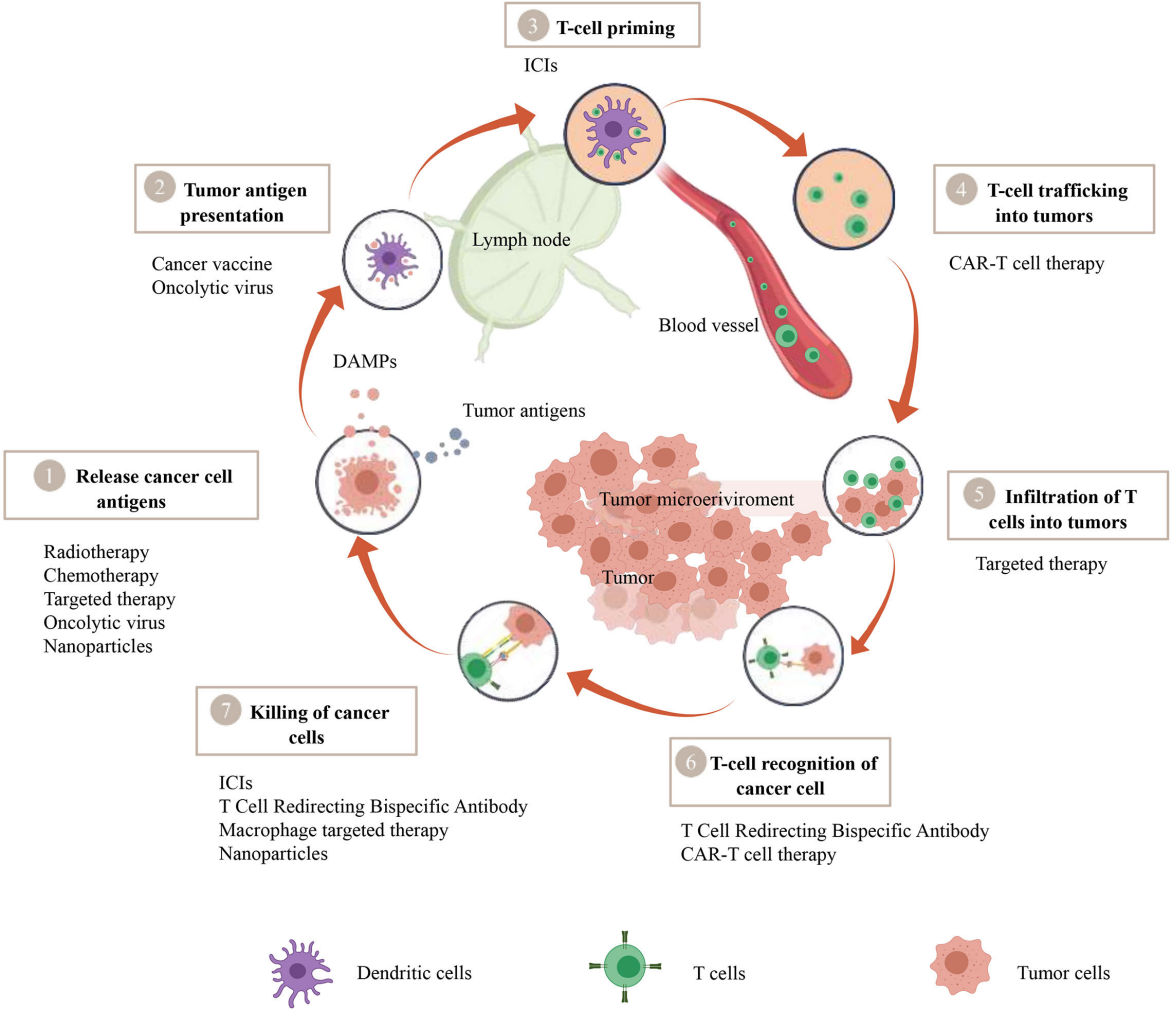
A therapeutic strategy to convert cold tumors into hot tumors based on tumor immune cycle. The cancer-immunity cycle encapsulates seven pivotal steps, with each one being integral to the overall mechanism. A malfunction or inefficiency at any juncture can potentially instigate the tumor to evade the immune response. Nevertheless, a wide array of therapeutic approaches such as Chimeric Antigen Receptor T-cell (CAR-T) therapy, T-cell Redirecting Bispecific Antibodies, cancer vaccines, oncolytic viruses, macrophage-targeted therapies, radiotherapy, chemotherapy, targeted therapies, and nanoparticle-assisted treatments manifest their potential to modulate this cycle, thereby amplifying the body’s defensive reaction against tumors.

Immune checkpoints encompass a group of receptors expressed by immune cells, facilitating the dynamic regulation of immune homeostasis. They hold particular relevance for the functioning of T cells. Among these checkpoints, PD-1 and its primary ligand PD-L1 find expression on T cells, tumor cells, and myeloid cells infiltrating tumors. The interaction between PD-1 and PD-L1 leads to CD8^+^ T cell exhaustion, a potentially irreversible state of dysfunction characterized by diminished or absent effector functions (including cytotoxicity and cytokine production), reduced responsiveness to stimuli, and altered transcriptional and epigenetic profiles (13, 14). Tumor cells exploit this interaction to establish immune tolerance. However, it also serves essential physiological roles, such as limiting autoimmune inflammation, preserving fetal tolerance during pregnancy, and preventing the rejection of transplanted organs (15). Immune checkpoint inhibitors function by blocking immune checkpoints, thus restoring the anti-tumor activity of CD8+ T cells. Immune checkpoint inhibitors function by blocking immune checkpoints, thus restoring the anti-tumor activity of CD8+ T cells. Any failures during the stages of T cell activation, homing, or infiltration into the tumor bed in the tumor immune process can result in inadequate T cell infiltration into the tumor core (**Figure 1**). This, in turn, leads to resistance to ICIs therapy.

### 3.1 Lack of tumor antigens

Tumor antigens can be categorized into two main types: Tumor-specific antigens (TSAs) and Tumor-associated antigens (TAAs) (16). TAAs are antigens that, while not exclusive to tumor cells, are present in normal cells and other tissues but are significantly elevated during cellular transformation into cancer. These antigens exhibit quantitative changes without strict tumor specificity. Although they can also trigger immune responses, the most crucial in activating immune responses are neoantigens, also known as TSAs. TSAs are antigens unique to tumor cells or present only in certain tumor cells and not in normal cells. This includes antigens produced by oncogenic viruses integrated into the genome and those arising from mutant proteins (17). In addition to mutations in DNA coding regions, gene fusions (18), mutations in non-coding regions (19), and alternative splicing (20) can also generate neoantigens. Loss of DNA damage response can lead to gene mutations, including mismatch repair deficiencies (dMMR) and microsatellite instability (MSI) (21). Currently, ICIs treatment has become the preferred therapy for advanced colorectal cancer with high microsatellite instability (22). Therefore, the recognition of TSAs plays a key role in activating T cells and promoting their infiltration into tumor tissues.

Tumor Mutational Burden (TMB) refers to the number of nonsynonymous single nucleotide mutations found in tumor cells. A high TMB implies more mutations, leading to the generation of more TSAs. Research over the past five years has shown that tumors with high TMB respond better to ICIs treatment than those with low TMB (23). McGrail et al. found that in cancers characterized by recurrent mutations, neoantigens are positively correlated with TILs infiltration (24). However, in tumors characterized by recurrent copy number variations, there is no correlation between TILs infiltration and the neoantigen load (24). Spranger et al. found no association between T cell infiltration and nonsynonymous somatic mutations (NSSMs) (25). These studies indicate that the lack of T cell infiltration cannot be solely explained by TMB.

### 3.2 Defective antigen presentation

Dendritic cells (DCs) are pivotal in the antigen presentation process. They play a critical role in initiating anti-tumor immune responses by capturing and processing tumor antigens, conducting cross-presentation, and activating naive T cells. There are multiple subgroups of DCs, including classical DCs (type 1 cDC1 and type 2 cDC2), plasmacytoid DCs (pDCs), inflammatory DCs, and Langerhans cells. Each subgroup plays a distinct role in immune responses (26). In tumor immunology, DCs are often activated by “danger signals” such as Damage-Associated Molecular Patterns (DAMPs), including ATP, HMGB1, Calreticulin (CRT), and the S100 protein family (27). Among the different DCs subtypes, cDC1s are particularly crucial in tumor immunity. Studies show that Batf3-knockout mice, which lack cDC1s, exhibit reduced TILs and decreased responsiveness to ICIs (28). Research on tumor-bearing mice indicates that cDC1s are essential for reactivating circulating memory anti-tumor T cells and responding to ICIs (29). Tumors can evade detection by DCs through various mechanisms, such as expressing the “don’t eat me” signal CD47 (30). Tumor cells also avoid exposing DAMPs, such as CRT, by expressing inflammatory molecules like A20 (31) in CRC, STC1 (32) in certain tumors, and glycosylated B7-H4 (33) in breast cancer. cDC1s cross-present antigens from dying tumor cells, which is fundamental in initiating anti-cancer CD8+ T cell responses. cDC1s express high levels of DNGR-1 (also known as CLEC9A), a receptor that binds to exposed F-actin in dying tumor cells and facilitates antigen cross-presentation. Tumor cells can inhibit this process by secreting extracellular proteins like sGSN, reducing the binding between DNGR-1 and F-actin, thus preventing cDC1s from activating CD8+ T cells (34).

HLA-I Loss of Heterozygosity (HLA-I LOH) is a significant mechanism of immune escape, with approximately 17% of tumors exhibiting HLA-I LOH (35, 36). TRAF3, a factor that inhibits NF-κB activity, negatively regulates the expression of MHC-I. Lower levels of TRAF3 are associated with better responses to ICIs) (37). Notably, MHC-I on the surface of Pancreatic Ductal Adenocarcinoma (PDAC) cells is degraded through autophagy. Inhibiting autophagy can restore MHC-I levels on the surface of PDAC cells. In mouse models of PDAC, combining autophagy inhibitors with dual ICIs enhances the immune response against the tumor (38). Therefore, increasing the expression of HLA-I on tumor cells’ surface could be a potential strategy for treating ‘cold’ tumors.

### 3.3 T lymphocytes are unable to infiltrate the tumor bed through the blood circulation

#### 3.3.1 Dysregulation of chemokines and cytokines

Chemokines in the TME mediate the recruitment of various immune cells, including T cells, thereby influencing tumor immunity and treatment outcomes. Dysregulation of chemokines within the TME often promotes tumor progression by altering the infiltration of immune cells. For instance, effector CD8+ T cells, Th1 cells, and NK cells can migrate into the tumor in response to chemokines like CXCL9 and CXCL10, facilitated by their shared expression of the CXC chemokine receptor 3 (CXCR3) (39). Enhancer of zeste homologue 2 (EZH2) and DNA methyltransferase 1 (DNMT1) reduce the presence of effector T cells in tumors by inhibiting the production of CXCL9 and CXCL10 by Th1 cells (40). In colorectal cancer, the polycomb repressive complex 2 (PRC2) similarly suppresses the production of these chemokines by Th1 cells, thereby diminishing the entry of effector T cells into the tumor (41). Additionally, the expression of CCL5 is associated with the infiltration of CD8+ T cells, while DNA methylation leads to reduced expression of CCL5, consequently decreasing TILs (42). Reactive nitrogen species (RNS) produced in the TME can also induce nitration of CCL2, impeding T cell infiltration (43).

Cytokines significantly impact tumor cell development and the treatment outcomes of ICIs. For instance, in urothelial cancer, combining Transforming Growth Factor β (TGF-β) blockade with ICIs therapy has been shown to promote T cell infiltration into the tumor core and elicit strong anti-tumor immune responses (44). Similarly, in colorectal cancer, inhibiting TGF-β increases the number of cytotoxic T cells, thereby inhibiting tumor metastasis (45). Additionally, Interferon γ (IFNγ), Interleukin-2 (IL-2), and Interleukin-9 (IL-9) also play crucial roles in the efficacy of ICIs treatment (46).

#### 3.3.2 Immune cell–mediated immunosuppression

Within the tumor microenvironment, tumor cells interact with various immune cells that have immunosuppressive functions, particularly regulatory T cells (Tregs), myeloid-derived suppressor cells (MDSCs), and tumor-associated macrophages (TAMs), playing a crucial role in the regulation of tumor development and progression (47, 48).

Tregs, initially identified as thymus-derived immunosuppressive cells among CD4^+^ T cells with a high expression of CD25 in mice (49), and later described in humans (50–52), gained recognition in the field of immunology. The discovery of Foxp3, a master regulator of Tregs, firmly established this population as an independent immunosuppressive cell lineage within CD4^+^ T cells (53–55). In current classification, Tregs are divided into natural/thymic and peripherally induced subsets, based on the sites of their development (56–58). Hence, it becomes imperative to distinguish Tregs from FOXP3-expressing conventional T cells in humans. In human studies, FOXP3-expressing CD4^+^ T cells are further categorized into three groups, depending on the expression of CD4, CD45RA, CD25, and/or FOXP3: 1) naive/resting Tregs, defined by CD4^+^CD45RA^+^CD25^low^FOXP3^low^ T cells; 2) effector/activated Treg (eTreg) cells, characterized by CD4^+^CD45RA^−^CD25^high^FOXP3^high^ T cells; and 3) non-Treg cells, identified as CD4^+^CD45RA^−^CD25^low^FOXP3^low^ T cells. Naive Tregs, initially displaying weak suppressive activity, have recently exited the thymus but remain quiescent in the periphery (59, 60). Upon TCR stimulation, naive Tregs exhibit vigorous proliferation and differentiate into highly suppressive eTreg cells. In contrast, non-Treg cells lack immunosuppressive functions and instead produce inflammatory cytokines, including interferon (IFN)-γ and IL-17 (61). Treg cells play a crucial role in dampening antitumor immune responses, particularly those directed towards tumor-specific effector T cells (62). These Treg cells are attracted to the TME, where they undergo local proliferation and differentiation into an activated subset with potent suppressive capabilities (63). Importantly, the presence of a high frequency of Treg cells and an elevated ratio of Treg cells to effector T cells, such as CD8+ T cells, within the TME is consistently associated with an unfavorable prognosis among patients with various cancer types (64, 65). Eliminating Tregs from the tumor environment can thus potentiate the anti-tumor immune response. Moreover, a lower CD8+T/Treg ratio has been identified as a poor prognostic indicator for the effectiveness of anti-PD-1 monoclonal antibody treatments (66). Post-immunotherapy scenarios where there is no appreciable increase in T effector cells coupled with a decrease in Tregs, or a surge in Treg cells within the tumor matrix, are often indicative of resistance to PD-1/PD-L1 monoclonal antibody therapies.

MDSCs, a diverse group of cells, are known to inhibit effector T-cell responses and foster the development of Tregs (67). The efficacy of immunotherapy is often reduced in the presence of the tumor microenvironment (68). These MDSCs are induced in immature myeloid cells by external agents such as tumor-derived factors, and they disrupt the production, proliferation, migration, and activation of MDSCs. MDSCs facilitate tumor invasion and metastasis, predominantly through factors like Indoleamine 2,3-dioxygenase (IDO), Arginase-1 (ARG1), Reactive Oxygen Species (ROS), IL-10, Inducible Nitric Oxide Synthase (iNOS), Cyclooxygenase-2 (COX-2), and Nitric Oxide (NO) (69). Additionally, MDSCs can attract Tregs to the tumor microenvironment to augment immunosuppression. Studies also reveal that inhibiting PI3K can synergize with immunocheckpoint inhibitors. In models where PD-1 monoclonal antibody treatment was ineffective, PI3K inhibition reduced MDSC circulation and recruitment, curtailed the production of immunosuppressive factors like IL-10 and TGF-β, and enhanced the secretion of inflammatory mediators such as Interleukin-12 (IL-12) and Interferon-Gamma (INF-γ), mirroring the combined inhibitory effects on CTLA-4 and PD-1 monoclonal antibodies (70, 71). These findings suggest that PI3K inhibitors could serve as potential adjunctive therapies with PD-1/PD-L1 antibodies, particularly in overcoming single-agent drug resistance. In the metabolic context, MDSCs derive energy from arginine metabolism, primarily through ARG1. Impairment of ARG1 activity can diminish the inhibitory capacity of MDSCs, thereby heightening the sensitivity of tumors to PD-1/PD-L1 antibodies (72).

TAMs, another influential cell type in immunotherapy, consist of M1-like macrophages that bolster anti-tumor immunity and M2-like macrophages that promote cancer. PD-1 expression is more pronounced in M2-like macrophages compared to M1-like macrophages (73, 74), and an increase in PD-1+M2-like macrophages correlates with advanced disease stages, hinting at their progressive accumulation in the tumor microenvironment (75). M2-like macrophages aid in tumor cell immune evasion through PD-1 and are activated by cytokines such as IL-4, IL-10, IL-13, or Colony Stimulating Factor 1 (CSF1), engaging in wound healing, tissue repair, and anti-inflammatory responses through cytokines including IL-10 (76). They also promote tumor invasion and metastasis via angiogenesis and remodeling of the extracellular matrix (77). Clinical studies have correlated high levels of TAMs with poor outcomes in various cancers (78). Targeting the C-C Motif Chemokine Ligand 2 (CCL2) and C-C Motif Chemokine Receptor 2 (CCR2) pathways in a lung adenocarcinoma mouse model led to reduced recruitment of M2 macrophages and inhibited tumor growth (79). Notably, using macrophage Colony Stimulating Factor 1 Receptor (CSF-1R) blockers reduces TAM frequency, increases IFN production, and enhances tumor cell response to drugs in pancreatic cancer models. When combined with PD-1 or CTLA-4 antibodies, and gemcitabine, CSF-1R blockers demonstrated increased efficacy (80).

In conclusion, the heterogeneous nature of inhibitory immune cells within tumors, influenced by chemokines, cytokines, and colony-stimulating factors in the tumor microenvironment, limits the effectiveness of PD-1/PD-L1 blockers when used alone. Resistance to immune checkpoint blockade may be indicated by factors such as the CD8+/Treg ratio, IDO, ARG1, CSF-1R, and the M1/M2 ratio. Addressing these indicators through combined therapeutic strategies could lead to more effective clinical outcomes and prognoses. The concurrent use of drugs targeting immunosuppressive cells, including IDO inhibitors, ARG1 inhibitors, PI3K inhibitors, and ICIs, has shown promise in clinical trials, particularly when used in dual combinations, offering manageable side effects and good clinical compliance. However, the effectiveness of combinations involving three or more such agents remains less explored.

#### 3.3.3 Vascular abnormalities and hypoxia

CD8+ T cells must enter the tumor core through the intratumoral vasculature (16). Their transport into tumor tissue depends on enhanced expression of adhesion molecules and chemokines in the tumor blood vessels, a process known as endothelial cell activation. However, poor activation of tumor blood vessels often leads to impaired transport of CD8+ T cells (81). Studies have shown that the absence of TILs is associated with overexpression of the endothelin B receptor (ETBR) (82). Tumor cells often promote angiogenesis by producing vascular endothelial growth factor (VEGF), which typically reduces the expression of vascular cell adhesion protein 1 (VCAM-1), thereby hindering T cell migration into the TME (83). Additionally, research indicates that Fas ligand (FasL, also known as CD95L) is selectively expressed in the vasculature of human and mouse tumors, whereas it is not expressed in normal vasculature. Expression of FasL enables endothelial cells to kill CD8+ T cells, but not Tregs (84). Tumors with poor vascularization, such as PDAC, due to their abnormal vascular structure and function, reduce the transport of immune cells and often exhibit high resistance to ICIs treatment (85).

Aberrant angiogenesis in tumors frequently precipitates conditions such as hypoxia, acidosis, and necrosis, subsequently impeding anti-tumor immune responses (86). Hypoxia, a defining characteristic of cancer, arises from a disparity between oxygen consumption and supply within the tumor milieu. This is attributed to the voracious oxygen consumption by rapidly proliferating tumor cells, coupled with inadequate oxygen delivery due to dysfunctional vasculature (87). The impact of hypoxia on TILs is complex and wide-ranging. Notably, hypoxia can stimulate the expression of CCL28 (88), VEGF (86), CD39 (89, 90), and CD73 (89, 90). These molecules are instrumental in angiogenesis and modulate T cell mobilization.

#### 3.3.4 Oncogenic pathway activation

In the field of oncology, the complex interplay between tumor cells and various signaling pathways is pivotal in shaping the tumor microenvironment (TME) and influencing therapy resistance. Tumor cells are known to hijack and modulate numerous pathways, notably including PKC, Notch, and TGF-β signaling. Recently, attention has also been drawn to the cyclic GMP–AMP synthase (cGAS)–stimulator of interferon genes (STING) and Siglec signaling pathways. These pathways play a critical role in sustaining a tumor-friendly microenvironment and fostering resistance to treatment, including multi-drug resistance.

##### 3.3.4.1 Protein kinase C signaling

In oncology, the role of PKC isoforms in the TME is increasingly recognized as critical in determining tumor behavior. PKC, a family of serine/threonine kinases, serves as a signal transducer for various molecules including hormones, growth factors, cytokines, and neurotransmitters. These molecules are key regulators of cell survival, proliferation, differentiation, apoptosis, adhesion, and malignant transformation (91–93). The interaction of ligands with receptors can activate phospholipase C, thereby upregulating activators of PKC signaling like diacylglycerol (DAG) and Ca^++^ (94, 95), subsequently modulating several molecular pathways such as Akt, STAT3, NF-κB, and apoptotic pathways. Interestingly, different PKC isoforms play varying roles in tumorigenesis and metastasis (94). For instance, PKC alpha demonstrates antitumor activity by influencing the polarization of TAMs within the TME (96). Conversely, PKC theta has been shown to suppress tumors by inducing immune suppression through CTLA4-mediated regulatory T-cell function (97–99). However, other isoforms, like PKC beta, are known to facilitate angiogenesis and invasiveness in certain tumors via the VEGF signaling pathway (100–102). The complexity of PKC signaling is further evidenced by its dual role as both a tumor promoter and suppressor, depending on the isoform and the context. This dual role presents both challenges and opportunities for therapeutic interventions.

Recent advancements in cancer treatment strategies have explored the modulation of PKC signaling, utilizing activators like Bryostatins (103) and Epoxytiglianes (104–106), as well as inhibitors such as CGP 41251, to counteract tumor growth and reverse multidrug resistance (107).

##### 3.3.4.2 PI3K-AKT-mTOR signaling pathway

The PI3K/AKT/mTOR signaling pathway, a pivotal regulator of cellular processes such as apoptosis, proliferation, movement, metabolism, and cytokine expression, plays a critical role in tumor development and resistance mechanisms, especially against PD-1/PD-L1 antibodies. Central to this pathway is the lipid phosphatase PTEN, a tumor suppressor that inhibits PI3K activity. PTEN deletion or mutation leads to the activation of PI3K/AKT and resistance to PD-1/PD-L1 in various cancers. PTEN’s expression is regulated through diverse mechanisms, including epigenetic silencing, post-transcriptional and post-translational modifications, and protein-protein interactions (108). PTEN’s downregulation is key in cancer progression, affecting cell energy metabolism, metabolic reprogramming of cancer cells, and influencing glucose uptake and protein synthesis. PTEN also plays a role in cell migration and senescence, with its loss leading to increased cell viability and promoting EMT and tumor cell migration (109). PTEN loss affects tumor immunotherapy, showing a correlation with resistance to immunotherapy, particularly impacting the tumor microenvironment (110). PTEN deficiency leads to downregulation of SHP-2, a negative regulator of JAK/STAT3 pathway, promoting tumor growth (111, 112). The loss of PTEN in certain cancers is associated with decreased T-cell function, increased VEGF production, and the release of anti-inflammatory cytokines, resulting in non-inflammatory tumors. Moreover, PTEN’s role extends to regulating PD-L1 levels, with its absence or constitutive expression of the PI3K/AKT pathway influencing PD-L1 expression (113, 114). This interaction affects PD-1/PD-L1 antibody responses and is subject to modulation by various intracellular signaling pathways, including RAS/RAF/MEK and JAK/STAT, influenced by IFN-γ released by immune cells. Selective inhibition of PI3K has shown to enhance the therapeutic effect of PD-1/PD-L1 and CTLA-4 antibodies in experimental models, indicating potential in reversing resistance to immunocheckpoint inhibitors (115). Further clinical studies are warranted to explore this possibility.

##### 3.3.4.3 TGF-β signaling

Transforming Growth Factor-Beta (TGF-β) plays a multifaceted role in the progression of cancer, affecting a variety of cellular processes including cell proliferation, angiogenesis, epithelial-to-mesenchymal transition, immune infiltration, metastatic dissemination, and drug resistance (116). Interestingly, TGF-β produced by tumor cells can alter the function of tumor-associated plasmacytoid dendritic cells (pDCs), particularly affecting their ability to produce Type I interferon, thereby impacting T cell recruitment (117, 118). This aspect of TGF-β signaling is crucial in understanding its role in excluding T cells from the TME.

Recently, the combined use of TGF-β blocking antibodies with PD-L1 antibodies has been proven effective in enhancing T cell penetration into tumors, boosting anti-tumor immunity, and leading to tumor regression (44). Additionally, TGF-β signaling plays a dual role in cancer progression. Initially, it acts as a tumor suppressor by inhibiting cell proliferation and inducing apoptosis (119). However, as malignancies progress, cancer cells exploit TGF-β signaling to create a favorable TME, activating CAFs, promoting angiogenesis, and suppressing anti-tumor immune responses (120–122). Given its complex role, the side effects of targeting TGF-β signaling in therapeutic interventions are also a concern.

##### 3.3.4.4 cGAS-STING signaling

Recent advancements in cancer research have increasingly focused on the cGAS-STING signaling pathway and its role in tumor progression. Analysis of The Cancer Genome Atlas (TCGA) database, which classifies 18 different types of malignant tumors, has revealed variations in the expression of key genes involved in the cGAS-STING signaling mechanism between normal and cancerous tissues. These include genes encoding cGAS (MB21D1), STING (TMEM-173), TBK-1, and IRF-3. Studies have found that these genes are significantly upregulated in nearly all cancer models, indicating a possible universal activation of cGAS-STING signaling in various cancer types (123, 124). Interestingly, some highly invasive tumors seem to rely on the cGAS-STING pathway to facilitate tumorigenesis, impacting cancer treatment approaches (125, 126). The NF-κB pathway, known for regulating cell proliferation, apoptosis, and survival, also plays a vital role in the inflammatory response. Its activation can contribute to inflammation, tumor development, and immune dysfunction. Chromosomal instability can lead to chronic inflammation by persistently activating the cGAS-STING pathway, which in turn enhances NF-κB function and promotes the progression of metastatic cancer cells (126, 127).

Furthermore, TCGA data analysis has demonstrated a negative correlation between STING expression levels in cancer and the infiltration of immune cells in various tumor models. This suggests that an increase in cGAS-STING signaling may predict poorer outcomes in cancer patients (123). Additionally, certain tumor cells promote brain metastasis by enhancing astrocyte-gap junctions through the expression of PCDH7 (composed of Cx43). These junctions transfer cGAMP from cancer cells to adjacent astrocytes, activating STING and triggering the production of TNF and IFN-α. These paracrine signals further activate NF-κB and STAT-1 pathways in metastatic brain cells, contributing to brain metastasis and resistance to lung and breast cancer therapies (128).

## 4 Therapeutic strategies for cold tumors

### 4.1 Dual ICIs

#### 4.1.1 α−CTLA−4 combined with α−PD−1/PD−L1

T cell activation requires two essential signals: the T cell receptor (TCR) and costimulatory pathways (129). Numerous costimulatory receptors have been discovered, which bidirectionally regulate T cell responses (130). Identified as the first molecule to deliver inhibitory signals, CTLA-4 is critical for concluding immune responses (131, 132). It negatively regulates T cell activation by, for instance, competing with CD28 for binding shared ligands B7.1 and B7.2 (133).

In clinical therapeutics, ipilimumab is seldom used in isolation. Physicians typically administer it in combination with nivolumab. While both CTLA-4 and PD-1 serve as immune checkpoints that inhibit T-cell activation via distinct mechanisms, their modulatory effects on immune response are uniquely characterized. Consequently, anti-CTLA-4 monoclonal antibodies may synergize with anti-PD-1/PD-L1 counterparts to potentiate tumor immunity. A growing body of research indicates that dual blockade of PD-1/PD-L1 and CTLA-4 exhibits enhanced antitumor efficacy in certain cancer types (134). Studies from CheckMate-069, CheckMate-067, and CheckMate-142 demonstrate that the combination of ipilimumab and nivolumab significantly improves clinical outcomes compared to monotherapy with either agent alone (135–137). Data from CheckMate-214, CheckMate-227, and CheckMate-743 further reveal superior treatment efficacy of the ipilimumab plus nivolumab regimen over standard targeted or chemotherapy approaches (138–140). To date, the U.S. FDA has approved the use of the ipilimumab and nivolumab combination for the treatment of melanoma, renal cell carcinoma, microsatellite instability-high/mismatch repair-deficient colorectal cancer, hepatocellular carcinoma, PD-L1 positive non-small cell lung cancer, and malignant pleural mesothelioma (134–140).

#### 4.1.2 α−PD−1/PD−L1 combined with ICIs

Emerging dual immune checkpoint blockade strategies, encompassing the combination of α-PD-1/PD-L1 with α-TIM-3, α-LAG-3, α-PVRIG, and α-TIGIT, remain in clinical trials and await regulatory approval. The ligation of TIM-3 to galectin-9 instigates apoptosis in Th1 cells via calcium flux (141). This dual inhibition, when applied to TIM-3 and PD-1/PD-L1 pathways, markedly augments anti-tumor immunity, as shown by slower tumor growth in murine models (142). Data from clinical trials suggest that this combined blockade does not increase adverse effects, although optimization of patient selection is warranted (143–145).

Extending the scope of ICIs, α-LAG-3, α-PVRIG, α-TIGIT, and α-Siglec-10, when used in concert with α-PD-1/PD-L1, enhance TIL functionality and concomitantly inhibit tumor growth (146–149). The RELATIVITY-047 phase 2/3 trial revealed a notable PFS advantage with relatlimab (α-LAG-3) plus nivolumab in late-stage melanoma (10.1 vs. 4.6 months; HR: 0.75), outperforming nivolumab alone (150). Additionally, COM701 (α-PVRIG) with nivolumab exhibited promising antineoplastic activity in phase 1 trial NCT03667716, inclusive of patients with prior ICI treatment (151). In phase 2 trial NCT03563716, tiragolumab (α-TIGIT) plus atezolizumab significantly improved both response rates (OR: 2.57, 95%CI: 1.07–6.14) and PFS (HR: 0.57; 95%CI 0.37–0.90) in PD-L1 positive NSCLC patients, compared with the control group receiving placebo and atezolizumab (152).

### 4.2 CAR-T cell therapy combined with ICIs

Chimeric Antigen Receptors (CARs) are multifaceted constructs, typically encompassing an extracellular antigen-binding domain, such as a single-chain variable fragment (scFv) targeting CD19, a hinge region to enhance antigen-receptor and tumor antigen interaction, a transmembrane domain for functional stability, and a T-cell activation domain (CD3 ζ) for primary signaling. Additionally, one or more intracellular co-stimulatory domains, like CD28/4-1BB, are included for secondary T-cell activation signaling (153). CAR T-cell activation is contingent on the presence of TAAs or TSAs. CARs’ unique ability to recognize diverse targets, including both protein and non-protein entities, on the cell surface, activates T cells without the necessity for antigen processing and presentation. This capability, bypassing human MHC constraints, positions CAR T-cell therapy as a revolutionary approach in T-cell therapeutic strategies, noted for its distinctive treatment characteristics (154).

CAR-T cell therapy’s hallmark is its non-reliance on Major Histocompatibility Complex (MHC) restrictions, coupled with an enhanced tumor-specific immune response, facilitated by the incorporation of co-stimulatory domains such as CD28, OX40, and 4-1BB. This attribute offers the potential to effectively target ‘cold tumors’, characterized by limited pre-existing T cell infiltration and a paucity of tumor antigens. A multitude of ongoing clinical trials are exploring CAR T-cell therapies against solid tumors, as elaborated in **Table 1**. Despite the direct tumor cell eradication capabilities of CAR-T cells, they remain susceptible to immunosuppression via immune checkpoints. Consequently, the synergistic approach of integrating ICIs with CAR-T cell therapy emerges as a promising treatment strategy (155, 156).

**Table 1:** Key clinical trials of immunotherapy combined with CAR-T cell/targeted therapy.

Study	Phase	Cancer type	Treatment	status	Start year
NCT04003649	I	GBM, *N*=60	IL13Ra2-CAR T+Ipilimumab	Recruiting	Dec, 2019
NCT03726515	I	GBM, *N*=7	CART-EGFRvIII+Pembrolizumab	Completed	Mar, 2019
NCT02366143	III	NSCLC, *N*=1202	Atezolizumab+bevacizumab +carboplatin + paclitaxel	Completed	Mar, 2015
NCT01984242	II	RCC, *N*=305	Atezolizumab+bevacizumab	Completed	Jan, 2014
NCT02420821	III	RCC, *N*=915	Atezolizumab+bevacizumab	Completed	May, 2015
NCT03434379	III	HCC, *N*=558	Atezolizumab+bevacizumab	Completed	Mar, 2018
NCT02501096	Ib/II	RCC, *N*=357	Lenvatinib+ pembrolizumab	Completed	Jul, 2015
NCT03517449	III	EC, *N*=827	Pembrolizumab+Lenvatinib	Active, not recruiting	Jun, 2018
NCT02853331	III	RCC, *N*=861	Pembrolizumab + axitinib	Active, not recruiting	Sep, 2016
NCT02684006	III	RCC, *N*=888	Avelumab + axitinib	Active, not recruiting	Mar, 2016
NCT03609359	II	GC, *N*=29	Lenvatinib+pembrolizumab (single-arm)	Completed	Oct, 2018
NCT02811861	III	RCC, *N*=1069	lenvatinib + pembrolizumab	Active, not recruiting	Oct, 2016
NCT02967692	III	melanoma, *N*=569	Spartalizumab + dabrafenib + trametinib (single-arm)	Active, not recruiting	Feb, 2017
NCT02752074	III	Melanoma, *N*=706	epacadostat + pembrolizumab	Completed	Jun, 2016
			pembrolizumab		
NCT02908672	III	melanoma, *N*=514	Atezolizumab+vemurafenib + cobimetinib	Active, not recruiting	Jan, 2017
NCT03082534	II	HNSCC, *N*=78	Pembrolizumab + Cetuximab	Active, not recruiting	Mar, 2017
NCT02734004	I/II	BC, *N*=264	Olaparib + durvalumab	Active, not recruiting	Mar, 2016

RCC, Renal Cell Carcinoma; HCC, hepatocellular carcinoma; EC, Endometrial Cancer; HNSCC, neck squamous cell carcinoma; BC, breast cancer; GBM, Glioblastoma.

### 4.3 CAR-NK cell therapy

A noteworthy attribute of mature NK cells in the field of adoptive cell therapy is their ability to retain functionality when transplanted into new environments with differing MHC expression patterns (157, 158). Unlike T lymphocytes, NK cells predominantly do not trigger graft-versus-host disease but instead exert a regulatory role (159). Advances in genetic modification techniques have shown that NK cells can be customized further, including the introduction of CARs and the knockout of inhibitory genes (160). These advancements enable NK cells from patients with hematologic malignancies to rapidly eliminate autologous tumor cells resistant to unmodified NK cells (161, 162). Preclinical studies on CAR-NK cells in xenograft mouse models have demonstrated *in vivo* activity comparable to CAR-T cells, yet with less cytokine release and improved overall survival rates (163, 164). The inaugural human study of CAR-NK cells has revealed promising anti-tumor responses without significant toxicities such as cytokine release syndrome and graft-versus-host disease (165). These positive outcomes lay the groundwork for further development of CAR-NK cells as a promising modality for cancer therapy (162).

Currently, CAR-NK cell-mediated immunotherapy is advancing rapidly, offering new therapeutic avenues for patients with malignant tumors. Despite extensive research in the field of cancer immunotherapy, the application of CAR-NK cells remains relatively limited to a variety of tumor models, primarily focusing on hematological malignancies (166). **Table 2** summarizes the clinical studies of CAR-NK cells in solid tumors.

**Table 2:** Clinical trials of CAR-NK cell-based cancer immunotherapy.

Study	Phase	Cancer type	Treatment	status	Start year
NCT03940820	I/II	Solid Tumor, *N*=20	Biological: ROBO1 CAR-NK cells	Recruiting	May, 2019
NCT03415100	I	Solid Tumor, *N*=30	Biological: CAR-NK cells targeting NKG2D ligands	Recruiting	Jan, 2018
NCT03931720	I/II	Malignant Tumor, *N*=20	Biological: BiCAR-NK/T cells (ROBO1 CAR-NK/T cells)	Recruiting	Mar, 2019
NCT03692663	I	Castration-resistant Prostate Cance, *N*=9	Biological: anti-PSMA CAR-NK cell	Recruiting	Dec, 2018
NCT04847466	II	Gastroesophageal Junction Cancers; Advanced HNSCC, *N*=55	Drug: N-803; Drug: Pembrolizumab; Biological: PD-L1 t-haNK	Recruiting	Dec, 2021
NCT03692637	I	Epithelial Ovarian Cancer, *N*=30	Biological: anti-Mesothelin Car-NK cells	Not yet recruiting	Mar, 2019
NCT03941457	I/II	Pancreatic Cancer, *N*=9	Biological: BiCAR-NK cells (ROBO1 CAR-NK cells)	Recruiting	May, 2019

### 4.4 T Cell redirecting bispecific antibody combined with ICIs

T cell-redirecting bispecific antibodies (BsAbs) represent a cutting-edge approach in immunotherapy, merging two monoclonal antibodies into a singular entity. Ingeniously engineered, these antibodies concurrently engage specific receptors on T cells, like CD3, and distinct tumor cell antigens. Central to their dual-specific functionality is the ability to directly steer T cells towards tumor cells, thus enhancing T cell-mediated identification and elimination of tumor cells. A distinctive feature of BsAbs-induced tumor cell lysis is its independence from conventional antigen recognition processes, which typically involve MHC class I or II molecules, antigen-presenting cells, or the necessity of co-stimulatory molecules (167).

Advancements in T cell-redirecting BsAbs for solid tumors lag behind those in hematological malignancies, in part due to a more limited range of available surface targets in solid tumors (167). Despite these challenges, four bispecific antibodies (BsAbs) have currently received FDA approval. These include Catumaxomab (Fresenius/Trion’s Removab^®^), which was withdrawn from the market in 2017, Blinatumomab (Amgen’s Blincyto^®^), Amivantamab-vmjw (Janssen’s Rybrevant^®^), and Tebentafusp-tebn (Immunocore’s Kimmtrak^®^) (168). In addition, there are still many BsAbs in the clinical evaluation stage for cancer treatment (**Table 3**). These agents signify progress in targeted therapeutic interventions, illustrating the evolving landscape of cancer treatment. However, even with their effectiveness, the T cells activated by these therapies can be rendered inactive by immune checkpoints. Consequently, a synergistic approach of ICIs in conjunction with T cell-redirecting BsAbs presents as a viable and potentially more effective treatment strategy.

**Table 3:** Bispecific antibody clinical trials ongoing.

Study	Phase	Cancer type	Treatment	status	Start year
NCT04506086	IV	B-precursor Acute Lymphoblastic Leukemia, *N*=45	Blinatumomab	Recruiting	Aug, 2021
NCT03415100	I	B-cell NHL, *N*=116	AZD0486 IV	Recruiting	Mar, 2021
NCT04844073	I/II	Advanced Cancer, *N*=228	MVC-101 (TAK- 186)	Recruiting	Mar, 2021
NCT04221542	I	Prostate Cance, *N*=461	AMG 509	Recruiting	Mar, 2020
NCT03564340	I/II	Recurrent Ovarian Cancer, *N*=690	REGN4018	Recruiting	May, 2018
NCT04117958	I	MUC17-positive Solid Tumors, *N*=58	AMG 199	Recruiting	Jan, 2020
NCT04104607	I	Castration-Resistant Prostatic Cancer, *N*=86	CC-1, PSMAxCD3	Recruiting	Nov, 2019

### 4.5 Cancer vaccine

‘Cold’ tumors, characterized by a dearth of tumor antigens, commonly exhibit immune evasion. Nonetheless, the use of cancer vaccines containing tumor antigens has shown efficacy in eliciting immune responses against such tumors (169). A range of cancer vaccines, designed to bolster the patient’s immune system, have received approval, including Tedopi, Ilixadencel, GVAX, and PolyPEPI101884 (170). Notably, Sipuleucel-T is the first FDA-approved cancer vaccine for metastatic castration-resistant prostate cancer (mCRPC), significantly prolonging patient survival (171). However, the therapeutic efficacy of cancer vaccines is often impeded by high PD-1 expression in effector T cells (172, 173). To address this, numerous phase 1 clinical trials have been initiated to investigate the combined use of cancer vaccines and immunoglobulins in cancer treatment, demonstrating their combined potential (174, 175). Ongoing clinical trials in this domain include NCT04300244, NCT03632941, KEYNOTE-603, and NCT03743298.

### 4.6 Oncolytic virus combined with ICIs therapy

Oncolytic viruses, encompassing both natural and genetically engineered variants, induce tumor cell lysis by selectively infecting and proliferating within tumor cells. Beyond their direct antitumor activity, these viruses also provoke a comprehensive, potent, and enduring anti-tumor immune response. This response is facilitated by the liberation of TAAs and additional DAMPs upon tumor cell demise (176). A significant aspect of oncolytic viral therapy is its systemic immunomodulatory impact, which extends its effects beyond the injection locus to distant tumor regions (176).

T-VEC, a modified herpes simplex virus, demonstrates augmented anti-tumor efficacy in treating unresectable stage IIIB-IV melanoma when used in conjunction with Ipilimumab, surpassing the results achieved with Ipilimumab alone (177). Furthermore, integrating a PD-1 inhibitor with oncolytic viral therapy significantly boosts its anti-tumor potency in glioma models (178). In the context of triple-negative breast cancer (TNBC), the response to ICIs typically remains suboptimal. Oncolytic viral treatment, however, renders TNBC more responsive to immune checkpoint blockade, successfully averting recurrence in a majority of the treated animal models (179).

### 4.7 Macrophage targeted therapy combined with ICIs therapy

TAMs, as key immune constituents in the TME, play an integral role in solid tumor development. These cells exhibit dual phenotypes: anti-tumoral (M1) and pro-tumoral (M2), with their behavior governed by their polarization state (180). TAMs significantly modulate immune responses by producing an array of cytokines and effector molecules. They suppress the function of T cells, B cells, NK cells, and dendritic cells, while simultaneously enhancing the roles of Tregs, T helper 17 cells (Th17), γδ T cells, and MDSCs. This multifaceted approach fosters an immunosuppressive milieu within the TME (181). Crucially, TAMs’ association with PD-L1 expression suggests that strategies combining ICIs with targeted TAM therapies could offer substantial therapeutic benefits (182, 183).

TAMs are pivotal in cancer treatment strategies. Targeting TAMs typically involves three approaches: 1) eradicating existing TAMs in the TME, 2) curtailing the recruitment of monocytes, and 3) reprogramming TAMs (181). TAMs are pivotal in cancer treatment strategies. Targeting TAMs typically involves three approaches: 1) eradicating existing TAMs in the TME, 2) curtailing the recruitment of monocytes, and 3) reprogramming TAMs (184, 185). In a phase 1b study (NCT02323191), a combination of the CSF-1R inhibitor emactuzumab with atezolizumab exhibited a superior Objective Response Rate (ORR) compared to controls (186). Additionally, in a separate clinical trial, the C-C chemokine receptor type 5 (CCR5) inhibitor maraviroc, used in tandem with pembrolizumab, demonstrated notable efficacy in patients with dMMR CRC (187).

### 4.8 Radiotherapy combined with ICIs therapy

Radiation therapy, employing ionizing radiation to directly destroy tumor cells, exerts multifaceted impacts on tumor immunity: 1) It can trigger immunogenic cell death (ICD) in tumor cells, culminating in the release of abundant DAMPs. These DAMPs, once phagocytosed by DCs, facilitate DC maturation (188). 2) Mature dendritic cells are capable of cross-presenting tumor antigens to CD8+ T cells, thereby initiating specific immune responses (189). 3) Concurrently, radiation therapy exhibits immunosuppressive properties, encompassing bone marrow suppression, the direct eradication of immune cells, upregulation of immune checkpoints, and the elicitation of immunogenic cytokines and chemokines (190–192). These immunoregulatory effects lay the groundwork for integrating ICIs with radiation therapy.

In certain cases, patients undergoing combined therapies exhibit spontaneous tumor regression beyond the irradiated zones, termed the ‘abscopal effect’ or radiotherapy’s distant impact. This phenomenon is largely attributed to radiotherapy enhancing the antigen presentation of tumor cells, thereby augmenting CD8+ T cell production. These cells then travel via the bloodstream to remote sites, influencing tumors outside the irradiated areas (193, 194). Contemporary research suggests that the abscopal effect can counteract immunosuppression and enhance the efficacy of ICIs (195–198). For instance, preliminary results from a phase III study on stage III unresectable NSCLC patients revealed that post-radiotherapy treatment with durvalumab significantly prolonged progression-free survival (PFS) compared to a placebo (199). Furthermore, another phase III study involving 799 participants demonstrated that, in patients with mCRPC previously treated with docetaxel, a combination of ipilimumab and radiotherapy markedly increased overall survival (OS) over placebo plus radiotherapy (200). Additional information on clinical trials combining radiotherapy with ICIs is detailed in **Table 4**.

**Table 4:** Key clinical trials of immunotherapy combined with radiotherapy.

Study	Phase	Cancer type (population,N)	Interventions and Combination	status	Start year
NCT02855203	I/II	ccRCC, *N*=30	Pembrolizumab+ SABR	Completed	Oct, 2016
NCT02904954	II	NSCLC, *N*=60	Durvalumab+SBRT	Completed	Dec, 2016
NCT02125461	III	NSCLC, *N*=713	Durvalumab + Chemoradiation therapy	Active, not recruiting	May, 2014
NCT02492568	II	NSCLC, *N*=92	Pembrolizumab + SBRT	Completed	Jul, 2015
			Pembrolizumab		
NCT02316002	II	NSCLC, *N*=51	Pembrolizumab + LAT (single-arm)	Active, not recruiting	Jan, 2015
NCT02444741	I/II	NSCLC, *N*=126	Pembrolizumab + SBRT	Active, not recruiting	Sep, 2015

ccRCC, Clear cell renal cell carcinoma; SABR, Stereotactic Ablative Body Radiosurgery; NSCLC, Non-small cell lung cancer; SBRT, Stereotactic body radiotherapy; LAT, Locally ablative therapy.

### 4.9 Chemotherapy combined with ICIs therapy

Chemotherapy drugs wield a bidirectional impact on the immune system during tumor therapy. Initially, they frequently induce systemic immunosuppression, evident through bone marrow suppression and lymphocyte depletion. Concurrently, these agents can eradicate specific immune cells, contributing to the reconstitution and establishment of a renewed immune system (201). The immunostimulatory actions of chemotherapy are manifested in several ways: 1) Augmenting antigenicity: Agents like cyclophosphamide, gemcitabine, platinum-based drugs, and taxanes boost the antigenicity of tumor cells. 2) Increasing susceptibility to immune assaults: This is primarily achieved by improving the visibility of tumor cells to the immune system. 3) Triggering ICD and antigen-specific responses: Anthracyclines, mitoxantrone, and oxaliplatin accomplish this by interacting with DNA replication and repair mechanisms (202). These pathways illustrate that chemotherapeutic drugs not only directly eradicate tumor cells but also engage in combatting tumors by stimulating and modulating the immune system.

Clinical trials integrating chemotherapy with immunotherapy have demonstrated considerable therapeutic success. In the phase III KEYNOTE-189 trial (NCT02578680), a regimen of pembrolizumab combined with pemetrexed-platinum agents yielded an ORR of 48.3%, markedly surpassing the 19.9% ORR of the placebo plus pemetrexed-platinum cohort. This combination therapy also significantly enhanced OS with a Hazard Ratio (HR) of 0.56 (95% Confidence Interval [CI]: 0.46-0.69) and PFS with an HR of 0.49 (95% CI: 0.41-0.59) (203). The phase III KEYNOTE-355 trial revealed that supplementing standard chemotherapy with pembrolizumab substantially improved PFS in metastatic TNBC patients with a combined positive score (CPS) of 10 or above (204). The KEYNOTE-021 (NCT02039674) trial found that pembrolizumab plus chemotherapy exhibited superior ORR (58% vs 33%) and PFS (median of 24.5 months vs 9.9 months; HR: 0.54; 95% CI: 0.35-0.83) compared to chemotherapy alone (205). Furthermore, in the phase III IMpower133 trial (NCT02763579), the addition of atezolizumab to carboplatin and etoposide significantly prolonged OS and PFS in small-cell lung cancer patients over the placebo with carboplatin and etoposide (206). Other clinical trials of chemotherapy combined with ICIs treatment are detailed in **Table 5**.

**Table 5:** Key clinical trials of immunotherapy combined with chemotherapy.

Study	Phase	Cancer type	Treatment	status	Start year
NCT02039674	II	NSCLC, *N*=267	Pembrolizumab + Chemotherapy	Completed	Feb, 2014
NCT02578680	III	NSCLC, *N*=616	Pembrolizumab+pemetrexed+platinum	Completed	Jan, 2016
NCT00324155	III	Melanoma, *N*=681	Ipilimumab+ dacarbazine	Completed	Aug, 2006
NCT00527735	II	NSCLC, *N*=334	Ipilimumab+paclitaxel+Carboplatin	Completed	Feb, 2008
NCT01285609	III	NSCLC, *N*=1289	Ipilimumab + chemotherapy	Completed	Jan, 2011
NCT03036488	III	TNBC, *N*=1174	Pembrolizumab+chemotherapy	Active, not recruiting	Mar, 2017
NCT02425891	III	mTNBC, *N*=902	Atezolizumab+Nab-paclitaxel	Completed	Jun, 2015
NCT02763579	III	ES-SCLC, *N*=503	Atezolizumab+carboplatin+etoposide	Completed	Jun, 2016
NCT02366143	III	NSCLC, *N*=1202	Atezolizumab+bevacizumab +carboplatin + paclitaxel	Completed	Mar, 2015
NCT02775435	III	NSCLC, *N*=559	Pembrolizumab+Chemotherapy	Active, not recruiting	Jun, 2016
NCT03043872	III	ES-SCLC, *N*=987	Durvalumab+tremelimumab +platinum-etoposide	Active, not recruiting	Mar, 2017
NCT02494583	III	GC, *N*=763	Pembrolizumab+chemotherapy	Completed	Jul, 2015
NCT02819518	III	TNBC, *N*=882	Pembrolizumab+chemotherapy	Active, not recruiting	Jul, 2016
NCT03134872	III	NSCLC, *N*=419	Camrelizumab+chemotherapy	Completed	May, 2017
NCT02853305	III	BLCA, *N*=1010	Pembrolizumab+chemotherapy	Completed	Sep, 2016
NCT02872116	III	GC, GEJ, OAC, *N*=2031	Nivolumab+chemotherapy	Active, not recruiting	Oct, 2016

TNBC, Triple-negative breast cancer; mTNBC, Metastatic triple-negative breast cancer; ES-SCLC, Extensive-stage small-cell lung cancer; GC, Gastric cancer; GEJ, Gastro-oesophageal junction cancer; OAC, Oesophageal adenocarcinoma; BLCA, Bladder urothelial carcinoma.

### 4.10 Targeted therapy combined with ICIs therapy

Targeted cancer therapy is predicated on creating potent inhibitors that specifically target molecular markers of tumor cells, aiming to effectively treat the cancer. The modes of action of this therapy span a range, including suppressing tumor cell proliferation, intervening in the cell cycle, promoting tumor cell differentiation, curbing metastasis, inducing apoptosis, and hampering tumor angiogenesis (207). Despite the considerable successes of many targeted therapies in clinical settings, the emergence of drug resistance in a significant number of patients represents a formidable challenge. Recent research has revealed that these targeted agents can trigger ICD in tumor cells, thereby bolstering the effectiveness of ICIs (207). Consequently, integrating targeted therapy with immunotherapy emerges as a novel and promising approach to surmount drug resistance and enhance therapeutic outcomes.

In the phase III IMspire150 trial (NCT02908672), the combination of vemurafenib, cobimetinib, and atezolizumab was compared against a control regimen of vemurafenib, cobimetinib, and placebo in patients with advanced or metastatic melanoma harboring the BRAF V600 mutation. The study revealed that the addition of atezolizumab significantly extended PFS to 15.1 months, compared to 10.6 months in the control group, with a HR of 0.78 (95% CI: 0.63-0.97, p=0.025) (208). In the phase 1/2 MEDIOLA trial, the efficacy of Olaparib combined with durvalumab was evaluated in patients with metastatic breast cancer with germline BRCA1 or BRCA2 mutations. This trial showed positive safety and disease control outcomes in 80% of patients after 12 weeks of treatment. These findings underscore the potential of integrating targeted therapy with immunotherapy in the treatment of certain cancers (209). Detailed information on additional clinical trials combining targeted therapy with ICIs is available in **Table 1**.

### 4.11 STING agonist combined with ICIs therapy

The accumulation of cytosolic chromatin fragments and micronuclei, a hallmark of malignant transformation in cancer cells, raises the likelihood of cytosolic DNA escape or DCs engulfing tumor-derived DNA (210). The cGAS-STING pathway, pivotal for cytosolic DNA detection, plays a crucial role in this context. Binding of cytosolic double-stranded DNA (dsDNA) to cGAS triggers the synthesis of cyclic GMP-AMP (cGAMP). This activation leads to the transformation of STING from a monomer to a dimer, facilitating its relocation from the endoplasmic reticulum to perinuclear microsomes. Subsequently, STING engages and phosphorylates TBK1, initiating a cascade that activates IRF3 and boosts IFN-I production (211–213). Additionally, the STING pathway enhances IFN-I through the NF-κB route (214). IFN-I, a multifaceted immune enhancer, significantly augments the functions of DCs, NK cells, and T cells (215). The cGAS-STING pathway’s integral role in linking innate and adaptive immunity underscores its potential as a target for cancer immunotherapy.

Initial clinical trials with Dimethyloxoxanthenyl acetic acid (DMXAA), the first STING agonist, were unsuccessful (216). Further research revealed that DMXAA specifically activates the mouse STING pathway, with minimal effects on its human counterpart (217, 218). Consequently, several natural and synthetic cyclic dinucleotides (CDNs), structurally and functionally akin to cGAMP, have emerged as promising STING agonists in cancer immunotherapy (219–221). However, these CDNs typically face challenges like limited transmembrane transport and reliance on intratumoral injection. Recent developments include novel STING agonists like diABZI and MSA-2, which offer systemic administration possibilities (222, 223). Moreover, manganese has been identified as a natural STING agonist, playing a significant role in anti-tumor immunity (224, 225).

In the context of combination therapy, the synergy of STING agonists with α-PD-1/PD-L1 antibodies presents a promising avenue. This approach simultaneously amplifies innate and adaptive immunity, effectively countering immunotherapy resistance. STING agonists enhance immune cell infiltration and amplify the functionality of APCs, NK cells, and T cells (226–228). Concurrently, α-PD-1/PD-L1 antibodies capitalize on the PD-L1 upregulation induced by STING agonists (227). Ongoing clinical trials involving combinations like ADU-S100 with spartalizumab, MK-1454 with pembrolizumab, and manganese with α-PD-1 have shown promising anti-tumor efficacy and tolerable safety profiles (229, 230).

### 4.12 Nanoparticles combined with ICIs therapy

Rapidly proliferating cancer tissues frequently form enlarged vascular endothelial gaps, with an average size of several hundred nanometers, to draw more nutrients from the body. Such gaps are generally not found in normal tissues. This phenomenon, known as the enhanced permeability and retention effect (EPR), allows nanoparticles of suitable size to infiltrate tumor tissues, while being restricted by the denser structure of normal tissues. This underpins the theoretical foundation for targeting tumor tissues with nanoparticles (231). Nanoparticles can amplify the efficacy of immunotherapy by inducing ICD in tumor cells (232). In melanoma mouse models, pH-sensitive liposomes equipped with a dual delivery system of doxorubicin hydrochloride and deferasirox have shown to enhance antigen presentation and T-cell infiltration, thereby augmenting their anti-tumor action (233). In glioblastoma multiforme (GBM) research, where drug delivery is constrained by the blood-brain barrier, BAMPA-O16B/siRNA liposomes have been able to effectively transport anti-CD47 and PD-L1 siRNA into intracranial GBM tumors in mice (234). Consequently, the strategy of using nanoparticles in combination with ICIs represents a promising avenue of research.

## 5 Conclusion and perspectives

In the evolving landscape of oncology, ICIs have emerged as a pivotal advancement. However, their efficacy is not uniform across all patient groups, highlighting a need for more nuanced understanding beyond the simplistic ‘cold’ and ‘hot’ tumor classifications. Intriguingly, some ‘hot’ tumors show responsiveness to ICIs despite a scarcity of CD8+ T cells, driven by NK cell-mediated immune responses. Conversely, ‘cold’ tumors often struggle with T cell activation and infiltration issues. Strategies that combine ICIs with other treatments such as radiotherapy, chemotherapy, CAR-T cell therapy, or targeted therapy are being investigated to transform ‘cold’ tumors into ‘hot’ ones, potentially increasing the efficacy of ICIs. Currently, many combination therapies fail to replicate these results in clinical settings. Currently, only a limited number of combinations, including α-PD-1/PD-L1 with chemotherapy, angiogenesis inhibitors, or α-CTLA-4, have received regulatory approval. The efficacy of most combinations remains confined to animal tumor models, underscoring the need for optimal preclinical models, with humanized patient-derived models offering more precise efficacy evaluations. However, combination therapies pose challenges such as increased immune-related adverse events (irAEs) and healthcare costs, and the risk of exposing patients to higher toxicities with inappropriate combinations. Optimizing administration regimens, including dosage, timing, and sequence, is crucial for the development of these therapies. Furthermore, the selection of suitable combination therapies and identification of predictive biomarkers for treatment response are still areas of active investigation. Liquid biopsy, by monitoring the dynamic immune landscape of the tumor microenvironment, offers a promising approach for real-time biomarker identification, guiding precision immunotherapy. Personalized combination therapies based on immune profiling and other predictive biomarkers, and a comprehensive framework integrating genomic, transcriptomic, immune, and microbiome profiles, could enhance patient selection for combination treatments. Particularly for patients with ‘cold’ tumors, α-PD-1/PD-L1 monotherapy often falls short of clinical benefits, necessitating personalized combinations to overcome drug resistance. In immune-desert scenarios, treatments such as radiotherapy, chemotherapy, and STING agonists can counter low immunogenicity-mediated immune tolerance by inducing immunogenic cell death and promoting antigen-presenting cell function. These combinations with α-PD-1/PD-L1 can simultaneously enhance multiple aspects of the cancer-immunity cycle, reshape the tumor microenvironment, and facilitate the transformation from non-inflamed to inflamed tumors. Additionally, the development of next-generation α-PD-1/PD-L1 drugs, including bifunctional or bispecific antibodies, could extend the indications for α-PD-1/PD-L1 therapies, allowing a broader range of patients to benefit from these advanced treatments.

## Author contributions

PO: Visualization, Writing – original draft. LW: Visualization, Writing – original draft. JW: Visualization, Writing – original draft. YT: Writing – original draft. CC: Writing – original draft. DL: Writing – review & editing. ZY: Writing – review & editing. RC: Writing – review & editing. GX: Writing – original draft. JG: Writing – original draft. ZB: Writing – original draft.
